# The Pain Intervention & Digital Research Program: an operational report on combining digital research with outpatient chronic disease management

**DOI:** 10.3389/fpain.2024.1327859

**Published:** 2024-02-02

**Authors:** Melanie Fu, Joanna Shen, Cheryl Gu, Ellina Oliveira, Ellisha Shinchuk, Hannah Isaac, Zacharia Isaac, Danielle L. Sarno, Jennifer L. Kurz, David A. Silbersweig, Jukka-Pekka Onnela, Daniel S. Barron

**Affiliations:** ^1^ Department of Psychiatry, Brigham & Women’s Hospital, Boston, MA, United States; ^2^ School of Medicine, University of Massachusetts, Wooster, MA, United States; ^3^ Department of Physiatry, Spaulding Rehabilitation Hospital, Charlestown, MA, United States; ^4^ Department of Biostatistics, Harvard T.H. Chan School of Public Health, Boston, MA, United States

**Keywords:** chronic pain, digital phenotyping, functional status, clinical operations, older (diseased) population

## Abstract

Chronic pain affects up to 28% of U.S. adults, costing ∼$560 billion each year. Chronic pain is an instantiation of the perennial complexity of how to best assess and treat chronic diseases over time, especially in populations where age, medical comorbidities, and socioeconomic barriers may limit access to care. Chronic disease management poses a particular challenge for the healthcare system's transition from fee-for-service to value and risk-based reimbursement models. Remote, passive real-time data from smartphones could enable more timely interventions and simultaneously manage risk and promote better patient outcomes through predicting and preventing costly adverse outcomes; however, there is limited evidence whether remote monitoring is feasible, especially in the case of older patients with chronic pain. Here, we introduce the Pain Intervention and Digital Research (Pain-IDR) Program as a pilot initiative launched in 2022 that combines outpatient clinical care and digital health research. The Pain-IDR seeks to test whether functional status can be assessed passively, through a smartphone application, in older patients with chronic pain. We discuss two perspectives—a narrative approach that describes the clinical settings and rationale behind changes to the operational design, and a quantitative approach that measures patient recruitment, patient experience, and HERMES data characteristics. Since launch, we have had 77 participants with a mean age of 55.52, of which *n* = 38 have fully completed the 6 months of data collection necessitated to be considered in the study, with an active data collection rate of 51% and passive data rate of 78%. We further present preliminary operational strategies that we have adopted as we have learned to adapt the Pain-IDR to a productive clinical service. Overall, the Pain-IDR has successfully engaged older patients with chronic pain and presents useful insights for others seeking to implement digital phenotyping in other chronic disease settings.

## Introduction

Chronic disease management, particularly in the context of older populations, poses a particular challenge for the healthcare system. Age-related physiologic changes contribute to overall medical comorbidity, an increased number of prescribed medications at higher doses, and greater vulnerability to adverse treatment outcomes, all of which complicate chronic disease management (1, 2). Functional status—an index of a patient's physical, emotional, and social wellbeing—is a critical measure in this context. Accurate assessment of functional status is especially important in value and risk-based models of healthcare delivery that bear the financial risk associated with patient outcomes, including decline in functional status, hospitalization, or relapse (3, 4). Risk-based reimbursement models therefore align patients' need for accurate, timely, and actionable monitoring of their health with the health system's need to help control costs. However, the difficulty of accurately assessing functional state in older patients is a well-established problem in medical practice (5). Real-time data could potentially enable timely interventions, manage risk, and promote better patient outcomes by predicting and preventing costly adverse outcomes such as emergency room visits, hospitalization, or surgery (6). The need for real-time measurement of functional status is especially acute in pain medicine.

Chronic pain, a complex health problem affecting up to 28% of U.S. adults and costing approximately $560 billion annually, presents significant consequences for healthcare systems (7). In individuals, the consequences of chronic pain accumulate over time, including decreased physical function, drug side effects such as addiction and dependence, and psychiatric symptoms such as depression and anxiety (8). Particularly in older patients, chronic pain management is challenging due to the interaction with other chronic diseases. One-fourth of all hospitalizations from adverse drug effects are linked to chronic pain treatments in older patients with medical comorbidities (1). Furthermore, accessibility of care can be an issue, especially for older patients with limited mobility or those residing in underserved areas who may struggle to find a pain provider.

While functional status is routinely gauged with patient-reported outcome measures (PROMs), these assessments come with their own set of challenges (5). Not only do they require substantial time investment from both patients and providers, but they offer only “intermittent, brief, and superficial cross-sectional examinations.” (9) Because PROMs rely on a patient's recall of prior experiences or health states over an extended period, the results may be skewed by their health state at the time of assessment (2). Healthcare inequities—whether socioeconomic, geographic, or simply lacking transportation to or from a clinical encounter,—may correlate with functional decline (10, 11). Remote, passive digital measures hold the promise of unobtrusively monitoring patient health in real-time.

Digital phenotyping has recently been defined as the “moment-by-moment quantification of the individual-level human phenotype in situ using data from personal digital devices,” such as smartphones (12, 13). In patients with spine disease, digital phenotypes of decreased mobility [measured with global positioning system (GPS) summaries] were associated with worsening pain scores (14). A more recent report, also in patients with spine disease, showed that voice recordings also predicted pain score (15). Therefore, while digital data show promise in tracking aspects of functional status, at present, no clinical model has successfully incorporated measures that are “continuous, precise, and reflect a patient's real-world functioning” in real-time (16).

Beyond the specific addition of clinical data, we emphasize the need for improved clinically relevant data that has bearing on clinical practice, that is, data that is shaped by and shapes the patient-clinician relationship. Previous work has shown that gathering and recording PROMs alone are not enough; provider interpretation and discussion of PROMs with patients is integral for PROMs to shape patient understanding of and motivation for their treatment plan (something elsewhere referred to as the patient narrative) (17). In other words, the ideal assessment of functional status would not only evaluate the patient's state of health but also serve as another useful layer of information to facilitate patient engagement with care conversations.

In this paper, we introduce preliminary findings from our Pain Interventional and Digital Research (Pain-IDR) Program. This innovative program integrates a research and clinical mission that seeks to develop new tools for measuring functional status and quality of care while providing standard care to chronic pain patients. Aligned with the overarching objective of digital phenotyping, we collect High-frequency Ecological Recordings of Mobility, Emotion, and Sociability, otherwise known as the HERMES phenotype. Our ambition is for the HERMES phenotype to provide “moment-by-moment quantification” quantification of a patient's functional state, aiding clinicians and patients in better understanding their health (18). Our report specifically focuses on the Pain-IDR's operational structure and our experience in the first 18 months of our rollout. We enumerate multiple obstacles and learning opportunities with the goal of helping others launch similar programs, of building capacity for and promoting the wider use of digital phenotyping in chronic disease management, and of improving healthcare delivery and clinical research.

## Methods

Broadly, this report outlines the Pain-IDR's two missions: the clinical mission, to offer standard-of-care management of chronic pain conditions; and the research mission, to develop digital tools to quantify functional status in older patients with chronic pain. From a clinical perspective, patients are asked to participate in a standard-of-care clinical evaluation and treatment plan. From a research perspective, study participants are asked to participate in a 6-month, longitudinal study that deploys Beiwe, a research platform for smartphone data, to collect active and passive digital measures that form the basis of the HERMES phenotype (described further below) (19).

We use two methodologies to report on the launch and iterative learning from the Pain-IDR: first, a narrative approach to describe the Pain-IDR's initial and adapted operational design, with key measures of clinical workflow; and second, a quantitative approach that describe the Pain-IDR's launch in terms of participant recruitment, the participant experience, and HERMES data characteristics. We provide a narrative report here in Methods, while we explain our quantitative approach in Methods and provide a report in Results.

### Pain-IDR operational design: a narrative approach

#### Initial operational design in Weymouth MA (*t* = 0 months)

The initial Pain-IDR pilot began in an outpatient community setting in Weymouth, MA, a rural setting that received referrals primarily from both in-network (Brigham and Women's Hospital) and out-of-network, local (South Shore Hospital) primary care physicians. The Weymouth location was a unique clinical setting and an ideal place to pilot the Pain-IDR: DB was the sole pain provider in the building and we had ample space, supportive staff who were willing to experiment, and flexibility of our schedule. This allowed us to learn and iterate through visit length, order, and clinical workflow.

To raise awareness of the Pain-IDR, we gave introductory talks to out-of-network, local providers (South Shore Hospital) and soon received many referrals for chronic pain management.

#### Eligibility

Patients scheduled at the Pain-IDR were referred through one of two methods: (1) Internally, through Epic-based electronic referrals, or (2) Externally, through online recruitment methods such as Rally and Research Patient Data Registry (RPDR). Eligible participants included (a) adults (>18 years old initially, and then >50 years old once we secured funding from the NIA) with a (b) chronic pain condition who (c) owned a smartphone and (d) were fluent in English. The criterion that patients simply have a chronic pain condition, we learned, was too broad: we received patients with, e.g., migraines, which was outside the domain of clinical expertise of the Pain-IDR. As we wanted to focus on patients with spine-related musculoskeletal pain, we modified our protocol to incorporate this specific criterion.

#### Participant recruitment

We measured our recruitment efforts at three phases: consent and onboarding, attrition, and completion. At each phase we evaluated whether demographic differences corresponded to differences in recruitment. Demographic data included patients' age, gender, sex, and ethnicity.

Initially, all patients entering the Pain-IDR clinic had a visit with the research assistant, which was the first time many of them heard about the study. However, as time passed, it became more efficient to have the research assistant and call center staff screen for patient interest preceding their initial appointment.

#### Virtual consent forms and recordkeeping

We developed virtual consent forms to better address patient needs. Initially, all patients entering the Pain-IDR clinic had a visit with the research assistant, which was the first time many of them heard about the study. However, as time passed, it became more efficient to have the research assistant and call center staff screen for patient interest preceding their initial appointment.

When we changed our practice location, we often had patients who wanted to meet virtually given mobility issues, age, or COVID-19 concerns. Thus, we built out a REDCap consent video and survey structure.

In order to better track of our study data, we developed a REDCap structure to facilitate troubleshooting and to learn more from the troubleshooting itself. This allowed the research assistant to monitor and catalogue common troubleshooting issues for review and future insight.

#### Visit schedules/onboarding

Broadly, there are two types of visits at the Pain-IDR: clinical and research. The goal of the clinical visit is to provide standard-of-care assessment and treatment. At the time of launch, initial clinical visits were 60 min and follow-up clinical visits were 30 min. The goal of the research visit is to educate and, when applicable, to consent and onboard participants to our research protocol. At the time of launch, initial research visits were 30 min and follow-up research visits were 15 min. Appointments were scheduled on a staggered workflow. A sample workflow for two consecutive initial visits (Patient A and Patient B) is below:

1) At 8 AM, Research Assistant sees Patient A for a 30 min initial research visit.
2) At 8:30 AM, Physician sees Patient A to begin 60 min initial clinical visit.
3) At 9 AM, Research Assistant sees Patient B for 30 min initial research visit.
4) At 10 AM, Research Assistant is done with Patient B's initial research visit, and Physician begins initial clinical visit for Patient B.

Staggering clinical with research visits allowed for optimization of clinic and staff time as well as reduced wait time and overall burden for our patients. However, as we progressed through the year, we also identified areas for improvement that led us to develop alternate workflows we further developed at Spaulding Charlestown (see below). Specifically, we learned that it was difficult to predict in advance how long a specific patient would take to consent and/or onboard onto the research platform. We learned that the time required per patient varied, in large part, on a patient's technological literacy. We define technological literacy as being able to perform certain functions using a smartphone, including downloading, opening, and closing an app; typing characters into fields; viewing phone notifications; and setting permissions in phone settings. Because technological literacy varied, we found that some patients ran over (when they had low literacy) or under (high literacy) our allotted time. For example, patients who had difficulty typing on their phones, were hard of hearing, or were unfamiliar with navigating phone settings often required some extra time to complete the onboarding process; conversely, patients who were very familiar with using their phones completed onboarding more quickly. Given this uncertainty, our clinical research visits often ran over or under time, both of which led to poor utilization of clinical space empty space in the schedule.

#### Visit length

Initially, visit length was 90 min (60 min for initial evaluation, 30 min for research visit) with follow-up visit length of 45 min (30 min clinical; 15 min research), however as clinical volume increased this was no longer tenable. We now have 40 min visit lengths with research visits interspersed as needed. This allows for a more efficient use of time.

#### Active and passive data collection

Once onboarded, study participants were asked to provide two types of data: passive and active data. Passive data included GPS location, accelerometer data, and smartphone usage statistics. Active data includes daily “micro-surveys”, administered in the Beiwe2 application platform (wherein the PROMIS-29 was broken into a total of 5 questions per day, one of them being a repeated measure of pain score) and daily audio journals (up to 3 min long). To continue participating in the study, we asked participants to contribute a minimum completion rate of 20 percent of active data (surveys) each month. All patients, regardless of study participation, were scheduled for routine clinical follow-up visits of 30 min duration. Participants were compensated monthly based on how much data were uploaded, as a way to promote patient engagement and monitor progress throughout the study. Our compensation model is presented in Supplementary Table S3.

#### Adapted operational design at Charleston Spaulding (*t* = 8 months)

After proving out that the Pain-IDR was clinically operational (i.e., that patients would consent to the 6-month research protocol and standard-of-care treatment), we moved to Spaulding Rehabilitation Hospital in Charlestown, MA. Moving the clinic location allowed us to provide a wider array of services to patients. Namely, Spaulding Charlestown offered the opportunity to offer fluoroscopic-guided and ultrasound-guided injections (e.g., epidurals, nerve blocks and ablations) to further measure shorter-term changes in functional status, pre and post-procedure. Spaulding further offered the opportunity to work with a group of like-minded pain providers, which was not available in Weymouth.

In Charlestown, patients are referred and scheduled at the Pain-IDR through one of two methods: (1) Internally, through the Spaulding Rehabilitation Hospital Physical Medicine and Rehabilitation referral pool; and (2) Externally, through online recruitment methods such as Rally and Research Patient Data Registry (RPDR). We continued our clinical inclusion criteria based on the pain location: spine pain, which includes neck or back pain; pain of the thigh, leg, foot, or hip; or buttock pain. Patients who have conditions that are unlikely spine pain are referred to other providers, such as abdominal, pelvic, or groin pain; face pain; ankle or knee pain; migraine; fibromyalgia or whole-body pain; knee pain; or chest pain.

Whereas in Weymouth, we were functioning solo with ample space, in Charlestown, we were embedded in a bustling, busy clinic with more limited space. Given these space constraints, we sought to further optimize our clinical workflow.

At Charlestown Spaulding, the clinic schedule transitioned to a 40 min initial evaluation with a 20 min follow-up visit. To solve the problem of varying, unpredictable visit length, we implemented our insights on scheduling in Weymouth and developed a schedule that no longer staggered clinical visits. Because it was not possible to predict a patient's technological literacy or even if they would consent to study participation, we performed the clinical initial visit before the research initial visit. The research assistant (RA) was present in clinic and was fortunate to have their own clinic room. If patients were interested in the research, the physician would refer the patient to the RA following the initial visit. Research participants were similarly directed to the RA following their clinical follow-up visit and the RA would hold a brief, research follow-up research visit. The ability to have a separate private room for research visits allowed the RA to personally check in with research participants, thereby establishing rapport which we feel was important in promoting longitudinal participation.

Based on the workflow, we implemented three layers of screening patients may go through to enter the Pain-IDR study: (1) Screening over telephone by call center staff; (2) Screening by research team during the Pain-IDR's weekly meeting; (3) Screening during the clinical visit, by discussing with a Pain-IDR clinician. In the telephone screening, for patients who meet research eligibility requirements (English-speaking, 50 years or older, owned their own smartphone), the call center staff sends an Epic message to the Research Assistant to notify them of the patient's interest in research. At the beginning of each week, the Pain-IDR also hosts a meeting to review that week's scheduled patients, screen new patients for research eligibility, and mark follow-up tasks for returning patients. Patients who may be eligible are flagged as recruitment candidates on the Epic scheduling system. Lastly, during a patient's initial visit, they are asked to electronically complete a PROMIS-10 and given a standard-of-care clinical evaluation. Following their initial visit, if the patient meets criteria for the study and expresses interest in the research, the research assistant will present the study to the patient. If the patient consents to the study, the research assistant will onboard the patient onto the study. Follow-up visits are of 20 min duration, hosted in the research assistant's clinic room.

While we began with in-person intakes and paper consents, we later learned that many patients required or preferred virtual intakes and electronic consent. Thus, we developed a REDCap survey that included a series of short videos and questions to check understanding. Our virtual education and consent tool allowed us to meet our patients' needs more comprehensively and efficiently.

### Pain-IDR launch: a quantitative approach

One of our goals was to evaluate the feasibility of conducting digital research in an older population with chronic pain and to implement the Pain-IDR's model of combined clinical care and research on a larger scale. We set recruitment goals in terms of participant recruitment and participant experience.

#### Participant compensation

We present summaries of how much participants were compensated in total (i.e., since launching the Pain-IDR). We further evaluated compensation totals for participants who completed 6 months and who either withdrew or were dismissed from the study. We were further interested in how many days participants engaged the study and broke this summary into two groups: those who completed the study and those who did not.

#### Participant experience

To understand and improve participants’ experience, participants were also asked to complete the User Engagement Scale Short-Form (UES-SF), as described in Supplementary Table S1 (20). This survey was performed digitally, using REDCap. A feedback survey was administered to participants upon completion of the study, which included Likert scale ratings as well as freeform answers. The Likert scale rating included responses 0 to 5, with responses including: Strongly disagree, Disagree, Neither agree nor disagree, Agree, Strongly Agree (all question and response options may be referenced in Supplementary Table S1). We consider participant feedback in two categories: technological (referring to the application) and content (referring to the PROMs survey questions).

#### HERMES data characteristics

We report on aspects of HERMES data characteristics that are relevant to the operational design of the Pain-IDR. Although we provide a description of type and collection frequency of the facets of the HERMES phenotype in Supplementary Table S2, a precise breakdown of the specific data types, trajectories, and models is beyond the scope of this paper and will be reported in a subsequent publication. Here, we focus on three primary data characteristics: data completion, participant compensation, and technologic difficulties experienced.

## Results

### Demographics and progress/ study population

**Figure 1:**
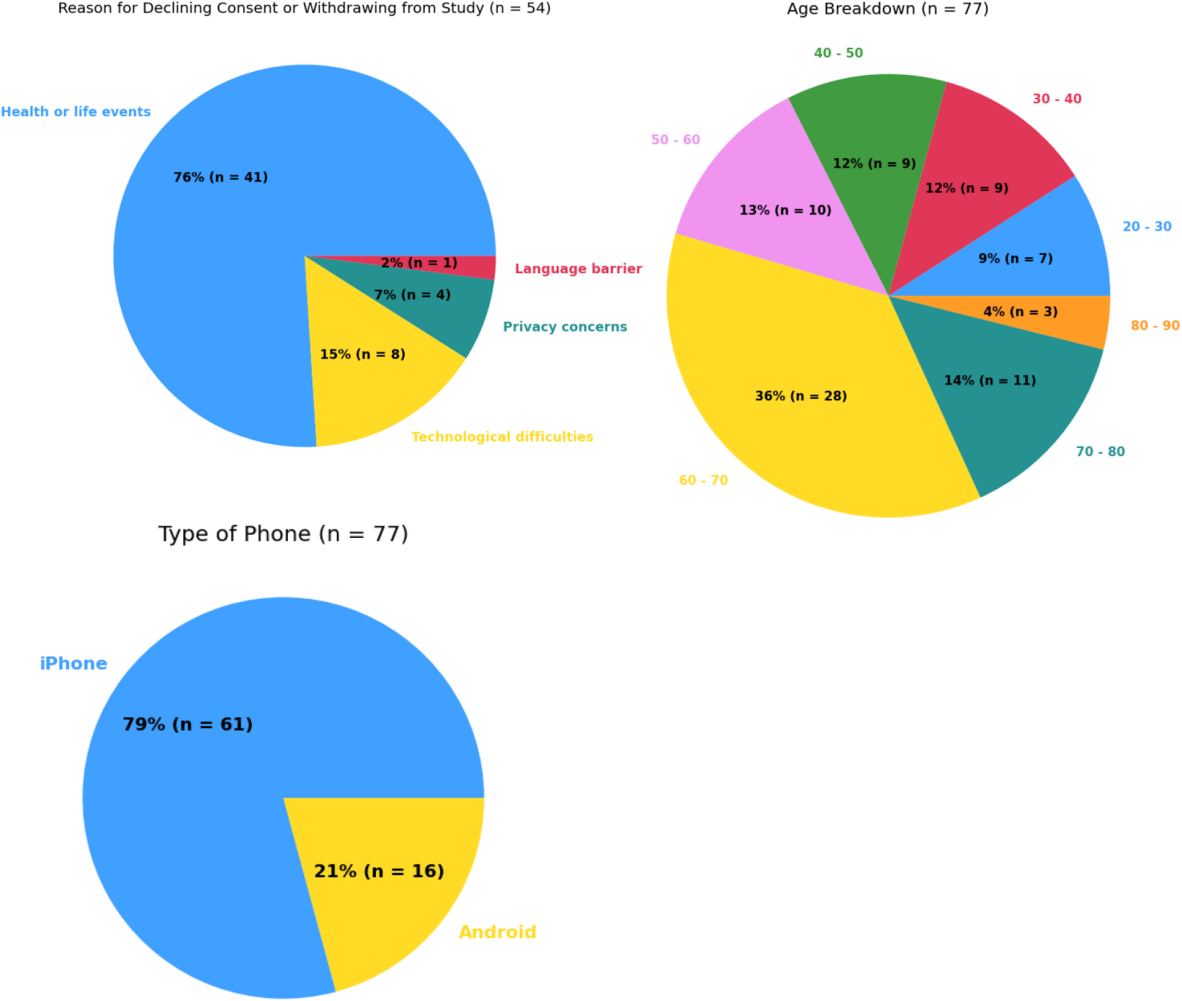
Summary statistics for most common reasons for declining consent or withdrawing from study (top left), age breakdown (top right), and types of smartphone usage (bottom left).

Within the Pain-IDR's first phase of recruitment, the average age of a consented study participant was 55. Further demographic information may be referenced in Table 1. The most common reasons for declining consent or withdrawing from the study were: lack of ability to participate in the study due to health or life events (76%), technological difficulties with the smartphone app (15%), privacy concerns (7%), and a language barrier (2%) (c.f. Figure 1, *n* = 54). One patient declined consent due to a language barrier; we thus incorporated the criteria of ability to read and complete surveys in English into our screening process. As the study progressed, we began to more proactively screen patients during a meeting at the beginning of the week (See Supplementary Figure S1 for a workflow schematic). There was no statistical difference between completers vs. non-completers in terms of age (*P* = 0.1, two-sided *t*-test) or gender (*P* = 0.86, Chi-square).

**Table 1:** Participant demographic information.

Participant Type	*n*	Mean Age	Std Dev Age	Range of Age (Min—Max)	Sex	Gender	Race
Total Participants	77	55.52	15.78	65 (20–85)	F: 51	F: 51 / MTF: 1	White: 61
					M: 26	M: 25 / FTM: 0	Black or African American: 6
							Asian: 2
							American Indian or Alaska Native: 1
							Other / Unknown: 7
Completed Study	38	58.82	13.28	55 (26–81)	F: 25	F: 25 / MTF: 1	White: 26
					M: 13	M: 12 / FTM: 0	Black or African American: 4
							Asian: 1
							American Indian or Alaska Native: 0
							Other / Unknown: 7
Not Completed	39	52.31	17.45	65 (20–85)	F: 26	F: 26 / MTF: 0	White: 35
					M: 13	M: 13 / FTM: 0	Black or African American: 2
							Asian: 1
							American Indian or Alaska Native: 1
							Other / Unknown: 0

F, female; M, male; MTF, male to female transgender; FTM, female to male transgender.

**Figure 2:**
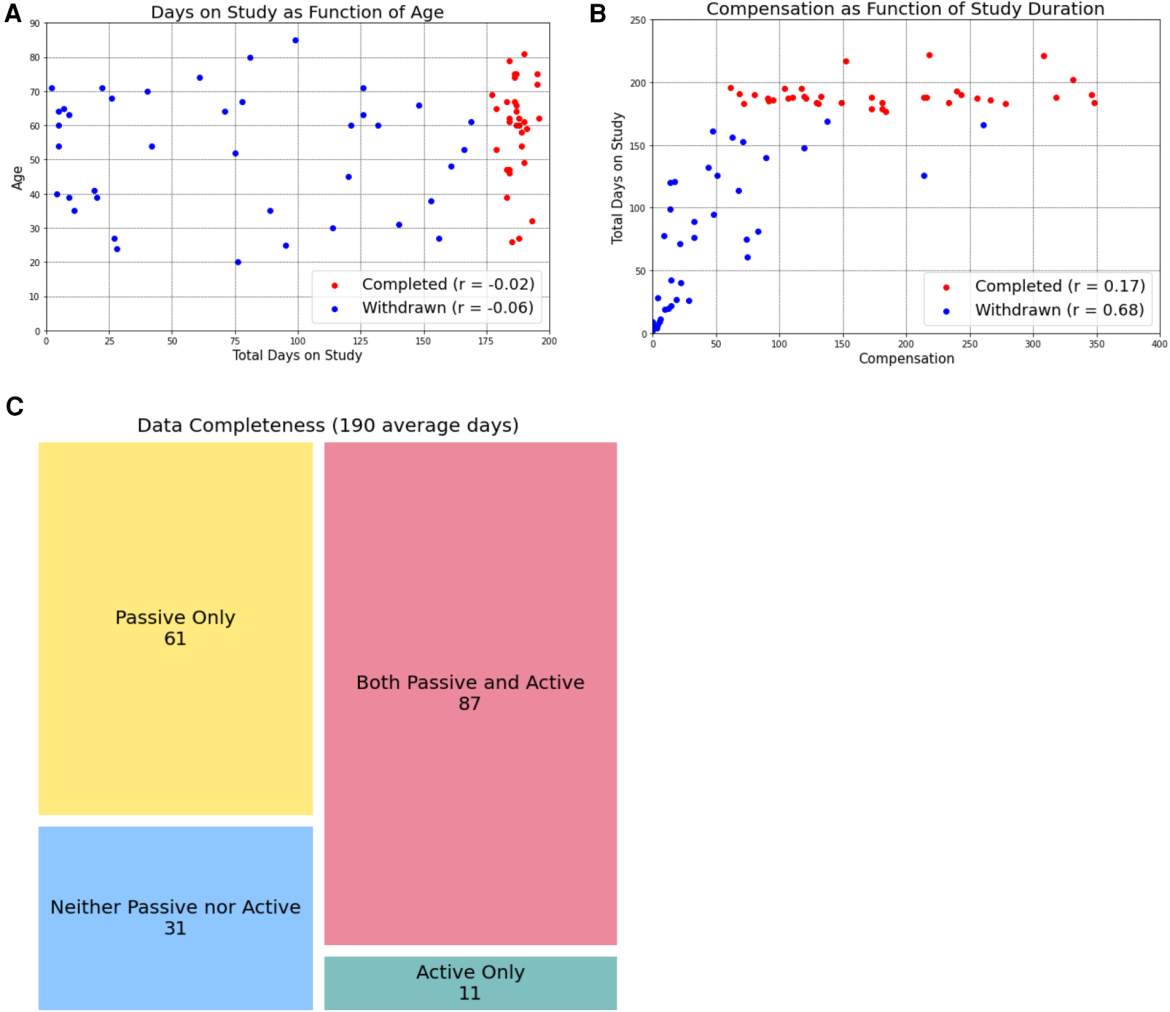
Age and compensation do not impact study completion. By separating participants into those who completed the study (red dots) and those who withdrew before the six-month timeline was complete, we learned that age (**A**) and compensation amount (**B**) were not related to study completion. However, in participants who eventually withdrew, the total days on the study was negatively correlated with age and positively correlated with compensation amount. Study completers uploaded an average of 190 days of data, of which passive data was more frequently collected. We present these data as data completeness (when data was present for a given day, (**C**).

Previous work has expressed concern about aging populations being less willing or able to participate in digital research due to lower technological literacy. We observed the opposite, with study completion not being impacted by age and, for participants who withdrew, total days on the study was negatively correlated with age (*r* = −0.06, Figure 2A). On average, participants who withdrew from the study before completion spent 72.77 days on the study.

### Participant compensation

Since launching the Pain-IDR, we have compensated participants a total of $8379.15. The total compensation was broken down into $6,708.97 for participants who completed 6 months on the study, and $1,218.25 for participants who withdrew.

The 77 participants who consented and were onboarded onto the study (including those who later withdrew), spent an average of 130.56 days on the study and were compensated an average of $109.84.

Participants who completed 6 months on the study had an average age of 58.82, spent an average of 189.87 days on the study, and were compensated an average of $177.09. Participants who withdrew or did not complete 6 months on the study had an average age of 52.31, spent an average of 72.77 days on the study, and were compensated an average of $44.32.

### Participant experience: user engagement scale-short form

On a Likert scale rating (from “Strongly disagree” to “Strongly agree”), study participants submitted an average score of 3.88 (*n* = 17) to the statement “I enjoyed using the Beiwe app and being a part of the study.”

Freeform feedback for improvement from participants can be divided into two main categories: technological comments about the application and experiences the PROMS survey questions used in Active data collection. Examples include:

“I liked the app when it worked. However, for most of the time the app did not display the surveys to fill out which resulted in my use being very inconsistent.”
“I thought the app design was too vague so you wouldn't be able to assess accurately. I found this frustrating but liked evaluating myself daily and liked the requirements of being positive.”

Experiences with the PROMs survey questions were largely positive, with some participants reported enjoying the feeling of “self-awareness” cultivated through the survey questions:

“I enjoy doing research studies and I really enjoy doing the belief for some reason it made me be aware of what I felt that day.”
“I enjoyed using the app. It was uncomplicated and straightforward. The study made me think more closely about my pain and what affected it.”
“I would say you've got a captive audience and can ask a lot more questions that get at pain and how it impacts the various facets of life. One thing that was never asked for example was if pain impacts sex life and libido. Just a suggestion.”
“The daily surveys were way too frequent. It made me disengage from the whole thing. Maybe if the questions changed or I could use my data in real time to make change it would be fine but it was way too repetitive.”

Outside the structured user engagement scales and freeform surveys, we discussed participant comments as a lab and found that one concern was whether the data collected reflected their day-to-day reality; that is, how much of a person's experience could a GPS and/or accelerometer tracing capture? We modified our training/onboarding materials to include information about how each data stream was intended to only capture a facet of each person's lived experience and that measures of mobility, emotion, and sociability were not intended to nor could they represent a person's full lived experience.

### HERMES data completion

The first IRB protocol we had approved did not have a minimum completion requirement for each month. After observing that some participants no longer responded to the micro-surveys a few weeks after being onboarded on the study, we realized we needed a structured, formal way to dismiss people from the study. We therefore amended our IRB protocol so that study participants with 1 month of data with less than 20 percent completion would be notified of this and asked to troubleshoot; participants with more than 2 consecutive months of data with less than 20 percent completion would be dismissed from study.

Additionally, we also developed a closed-loop learning system (further described in Discussion) in order to touch base with participants regularly to promote engagement. Check-ins with participants happened during both monthly follow-up visits as well as during biweekly (twice a week) data checks completed via phone call or email, performed by the research assistant to ensure all participants were above the 20 percent minimum completion rate.

Data completion provides a measure to assess the feasibility of longitudinal data collection over a 6-month period. Across participants who completed a full 6 months of the study, the mean number of days with either active or passive data was 190 days, with a mean completion rate of 84%.

However, when we break the completion rate into active and passive data, we see differences in the ease of collection. The active data completion rate (defined as days with any active data) was 51%, while the passive data completion rate (defined as days with any passive data) was 78%.

### Troubleshooting

We fielded 49 troubleshooting incidents, and the predominant issues reported were as follows: Survey Not Showing (6 cases, 12%): Participants encountered difficulties with surveys not appearing as expected. Data Uploading (16 cases, 33%): A significant number of participants faced challenges with the application that interfered with data collection. Mic Issues (2 cases, 4%): In a few instances, microphone-related problems were experienced when patients tried to collect audio data. Miscellaneous (15 cases, 31%): Miscellaneous issues accounted for a notable portion of the reported troubleshooting issues that didn't prohibit data collection but made the Beiwe2 interface difficult for patient use. Conflated (Mic/Data) (2 cases, 4%), Conflated (Misc/Data) (1 case, 2%), and Conflated (Survey/Data) (1 case, 2%) were noted to demonstrate the incidents in which the troubleshooting began with one category and ultimately resolved in another category. Finally, N/A (6 cases, 12%): These incidents were categorized as not needing specific troubleshooting. Further information about Troubleshooting can be referenced in Supplementary Table S5.

In response to these concerns, we developed a “Study Onboarding Tips” sheet to proactively prepare participants for potential challenges. This resource provides troubleshooting guidance for these most identified instances, such as survey visibility and data upload issues, as well as resources for participants to contact the Research Assistant for assistance with these problems.

## Discussion

We report on the Pain-IDR's design, launch, and preliminary results as a combined outpatient pain management and digital research clinic. Our overarching goal was to test the feasibility of deploying digital devices in a chronic disease management setting, specifically in older patients with chronic pain. In both rural and urban clinical settings, our preliminary findings are optimistic: participants engaged the digital research over the space of 6 months. To help others who might want to launch a similar clinic, we discuss what we have learned in the general context of digital phenotyping studies, and in the specific context of our research clinic design and operations.

### Digital phenotyping study challenges

Digital phenotyping studies must manage multiple challenges in multiple domains. From the perspective of participants, these include concerns about privacy, data accuracy, and overall study burden (21). These concerns held true in our population of older patients with chronic pain. For example, during the onboarding process, participants must enable location settings to allow accelerometer and GPS data collection and may be worried about privacy and data security. Privacy is an understandable concern that we take seriously and, likely because this value was communicated to our participants, we were able to conduct our study. Through developing robust supplementary participant education materials that explained the study's time commitment and requirements and specifically address any patient concerns about privacy or technology, we ensured that participants were satisfactorily informed to consent to the study. As we learned that individuals were willing to participate in the study, but some needed additional technological support, we made a research assistant available as a point of contact for any participant questions or concerns, allowing a continuous channel of communication and support as participants moved through the study. Therefore, the research assistant not only helped organize and execute the study design but also served as what has been called a participant “digital navigator” (22, 23). Consistent with Wisniewski (23), the digital navigator role provided non-clinical support around Beiwe use and, further, supported the therapeutic alliance as a valuable team member. In the future, we would like to define ways to improve this interaction and therapeutic alliance.

Another concern participants shared was whether the data collected in reflected their day-to-day reality. This concern is shared by some in the clinical and scientific community to varying degrees: one (perhaps extreme) view questioned the long-term goals of digital phenotyping, specifically as an exercise in “epidemiological surveillance” aimed at “pathological omniscience.” (24) Meanwhile, the complex, clinical realities of chronic disease management are such that any improvement in measurement—especially of data we already believe are clinically relevant such as mobility, emotion, and sociability—represent welcome additions to the clinical ecosystem; that is, if we can define and validate ways to bring these measures to bear to benefit clinical decision and patient outcomes. Far from a nifty data science exercise, the promise of digital phenotyping is to improve clinical decision and to benefit patients.

Beyond concerns about privacy and data accuracy, data that require an investment of time and energy (i.e., active data) remain a challenge to participants who are already struggling to manage a chronic disease, especially over an extended time horizon. Even when offered financial incentives (which are “notoriously difficult to do well” (21), participants often struggle to stay engaged in survey completion over long periods of time. Financial incentives—whether to compensate, how much, and how often—remains an unanswered question in the digital phenotyping literature. Past studies largely did not involve compensation (4, 25, 26) or additional reminders or support for smartphone application use (4). We are aware of the larger conversation that study incentives, such as monetary payments, may limit the generalizability of studies (27), however made the decision to provide financial incentives to participants given the protracted timeframe of 180 days (longer than most all previous trials). We chose a prorated, increasing compensation model with the goal of incentivizing participants to complete the full 180 days and offered on-going check-ins and technical support to further promote participation. However, as show in Figure 2B, several participants completed 6 months on the study at a 20% completion rate (the minimum allowed), despite having the potential to earn higher compensation. These results suggest that financial incentives did not promote engagement in the way we had hoped however we do note that this low completion rate is likely in line with what could be expected in the real-world clinical setting, where we hope such a data collection instrument would benefit patients. While further work is needed to better understand participant motivations for engagement, is it clear that active data collection remains a barrier to this type of longitudinal study design, and, by extension, to longitudinal measurement of functional status in chronic disease management.

As a corollary to the problem of minimum participation, we further observe that while we considered “study completion” as 6 months (180 days) of data collection many of our participants who did not fully complete the study provided more days of data than participants who did complete the study. This can be observed in Figure 2B, where many participants who did not complete the study (and are therefore labeled with blue dots) received a greater total compensation amount than participants who did complete the study (red dots). Because compensation amount reflects the amount of data received from participants, this indicates that even though they did not complete the full 180 days of the study, we were still able to collect a large amount of data from them. Such an observation has encouraged us to rethink how we define “study completion,” framing the term less in terms of a hard number of days participated and instead in terms of how much data we have available for analysis. We anticipate that, in the real-world clinical practice, the amount of data collected would be a more valuable metric than simply the number of days a participant was enrolled in a program: it is the data uploaded that could define functional status and guide clinical decision.

Our experience with missing data is very much in line with past studies that deployed Beiwe, albeit with variations in study duration, data collection type, sample size, and adherence (see Supplementary Table S4). Overall, data collection suffers from longitudinal design; that is, the longer participants are asked to respond to daily questions, the less likely they are to supply them over an extended horizon. An outpatient study of 29 to 30 days in 13 patients with major depressive disorder found 77.78% adherence (28). A study with mean duration of 154 days in 14 participants with major depressive disorder, schizophrenia, or bipolar disorder wore a sensor patch and underwent passive accelerometer and GPS data collection from the Beiwe app had a mean duration of 154 days and found 58% of accelerometer data and 46% of GPS data collected (26). In a study that followed 95 patients recovering from cancer surgery for a mean of 131 days, full-length Short Form-36 (SF-36) surveys administered at 4, 12, and 24 weeks and daily SF-36 micro-surveys had response rates of 76% and 34% respectively (2). And finally, a Beiwe study of 105 participants spine disease sampled active and passive data over the space of 94.5 days and reported a daily survey response rate of 43.4% (14). Our response rate is very much within the range of past Beiwe studies, especially given our large population across an extended time horizon of 180 days.

Overall, our results highlight the allure of passive data collection to define functional status. While active data requires participant engagement (i.e., time and work), passive data represents the ability to define measures that are “invisible and labor-free.” (6) There are reports of passive data being associated with significant battery or storage drain in participant phones, participant phone settings being incompatible with the application, and other technological difficulties (21). While we encountered these challenges, we found success in developing a short learning cycle (further described below) to quickly address these issues as they arose. Future work from our group will focus on determining which passive data features capture clinically relevant aspects of functional status.

### Research clinic design: a learning system approach

A perhaps predictable (by others) but unanticipated (by us) lesson from the Pain-IDR launch was the need for routine, structured meetings to organize incoming information, discuss improvements, and execute on those improvements in closed-loop communication cycles. In other words, to successfully deploy our digital phenotyping study within an outpatient pain clinic, we needed what operational science experts call a learning system (29). The goal of a learning system, Bohmer et al. write, is to “create a rich data stream, analyze and test insights, make redesign decisions, rapidly implement planned changes, and close the loop by checking reliability and effectiveness.” (29) We implemented specific time in our weekly lab meeting to review research participant data collection and implemented a Tableau desktop to allow us access to up-to-date data collection. We have also implemented reflection sheets for our clinical and research staff where they can note observations from the day and identify action items to test and act on; these are reviewed weekly. The weekly timeframe to hold ourselves accountable for tasks thereby creating what Tony Fadell calls a “heartbeat” for our research clinic (30). In contrast to simply maintaining the status quo, learning is difficult and Bohmer writes that the ability to learn must be “embedded in the organization's structure and internal processes at every level, and reinforced through the culture and behavior of staff, including what leaders say and do.” (29) Paradoxically, although learning could perhaps be the most rapid during in-person clinical evaluations, the clinical environment provides unique challenges, where the day's schedule and motivations are not always aligned with a learning approach. We continue to learn how best to learn. At the participant level, we are working to build out the capacity for patients to submit “tickets” to notify staff of questions or concerns, which would allow standardized aggregation of common problems or concerns, permitting such a learning cycle approach.

### Strengths & limitations

The primary strength of our report is that we have shown that longitudinal data collection is successful in both rural and urban-based clinical settings, with real-world patients who are being actively treated for chronic pain conditions. We deliberately left our inclusion criteria broad in attempt to best represent whomever walked through the door. Because of this effort, we feel our experience will be valuable to other clinical settings in other geographies. Of course, there are several limitations to this work. Firstly, while the smartphone app format presents a more accessible way for patients to take symptom surveys and upload meaningful mobility measures, accessibility limitations remain: the app still requires participants to own a smartphone, have some degree of technological literacy and familiarity with using a smartphone app, be able to read and speak English, have an email address, and connect to Wi-Fi. Secondly, while the app collects useful data about symptoms and mobility, emotion, and sociability, the app's data collection is still to a certain extent contingent on the participant's willingness and/or ability to use the app. Participants who withdrew from the study cited lack of ability to participate in the study due to health or life events as the primary reason for withdrawal, in the future, we will consider what types of patients may end up being excluded from digital health studies, and consider how to make digital health measures more accessible to patients who struggle with complex health events or barriers to access. Additionally, there may be technological difficulties as previously described: a participant's phone may not be compatible with or may keep rejecting smartphone app permissions, or there may be other technological difficulties that are unpredictable or out of our control. Future opportunities for research thus include improving the smartphone application interface and the survey questions used for active data collection.

## Conclusion

We present preliminary results from the Pain-IDR, a novel hybrid digital research and outpatient chronic pain program. We show that older patients with chronic pain conditions are amenable to digital health data collection using a smartphone and remain engaged throughout a six-month study period. We further describe different clinic designs in both rural and urban clinical settings and identify elements of a successful embedded clinical research setup and how to converge on that setup more effectively by adopting a learning system approach.

Chronic pain and chronic disease management are costly, in large part due to the complexity of disease and difficulty to measure and thereby detect declines in functional status. The shift to value and risk-based reimbursement models will require accurate measures of chronic disease state—both to form the model and to detect changes in disease state that, acted on in a timely and effective manner, could reduce the costs and impact of negative health outcomes such as relapse or hospitalization. The Pain-IDR has deployed digital devices as a first step to measure functional status in older patients with chronic pain, we present an early success that digital devices can be implemented in both rural and urban outpatient clinical settings. Future work will report on how well the HERMES digital phenotype tracks functional status.

## Data Availability

The datasets presented in this article are not readily available because This is an operational report. We will analyze and report on Beiwe data at a later date. Requests to access the datasets should be directed to Daniel Barron dbarron2@bwh.harvard.edu.
