# Supplementary material for: The Pain Intervention & Digital Research Program: an operational report on combining digital research with outpatient chronic disease management
Source: Front Pain Res (Lausanne). 2024 Feb 2;5:1327859. doi: 10.3389/fpain.2024.1327859 (PMC10869590; doi:10.3389/fpain.2024.1327859)
Supplement: Supplementary file 1 [file Datasheet1.docx]

**Pain Intervention and Digital Research: an operational report on combining digital research and outpatient chronic disease management**

Melanie Fu^1,2^, Joanna Shen^3,4^, Cheryl Gu^3,4^, Ellina U. Oliveira^3^, Ellisha Shinchuk^3^, Hannah Isaac^3^, Zacharia Isaac^3^, Danielle Sarno^1,3^, Jennifer Kurz^3^, David Silbersweig^1^, Jukka-Pekka Onnela^4^, Daniel S. Barron^1,3^*

1. Brigham & Women’s Hospital, Boston, MA, USA

2. University of Massachusetts, Wooster, MA, USA

3. Spaulding Rehabilitation Hospital, Charlestown, MA, USA

4. Department of Biostatistics, Harvard T.H. Chan School of Public Health, Boston, MA, USA

*Corresponding Author

**Supplemental Figure.** Adapted workflow of the Pain-IDR Program, based on our learning cycle approach and feedback from the research and clinical team.

**
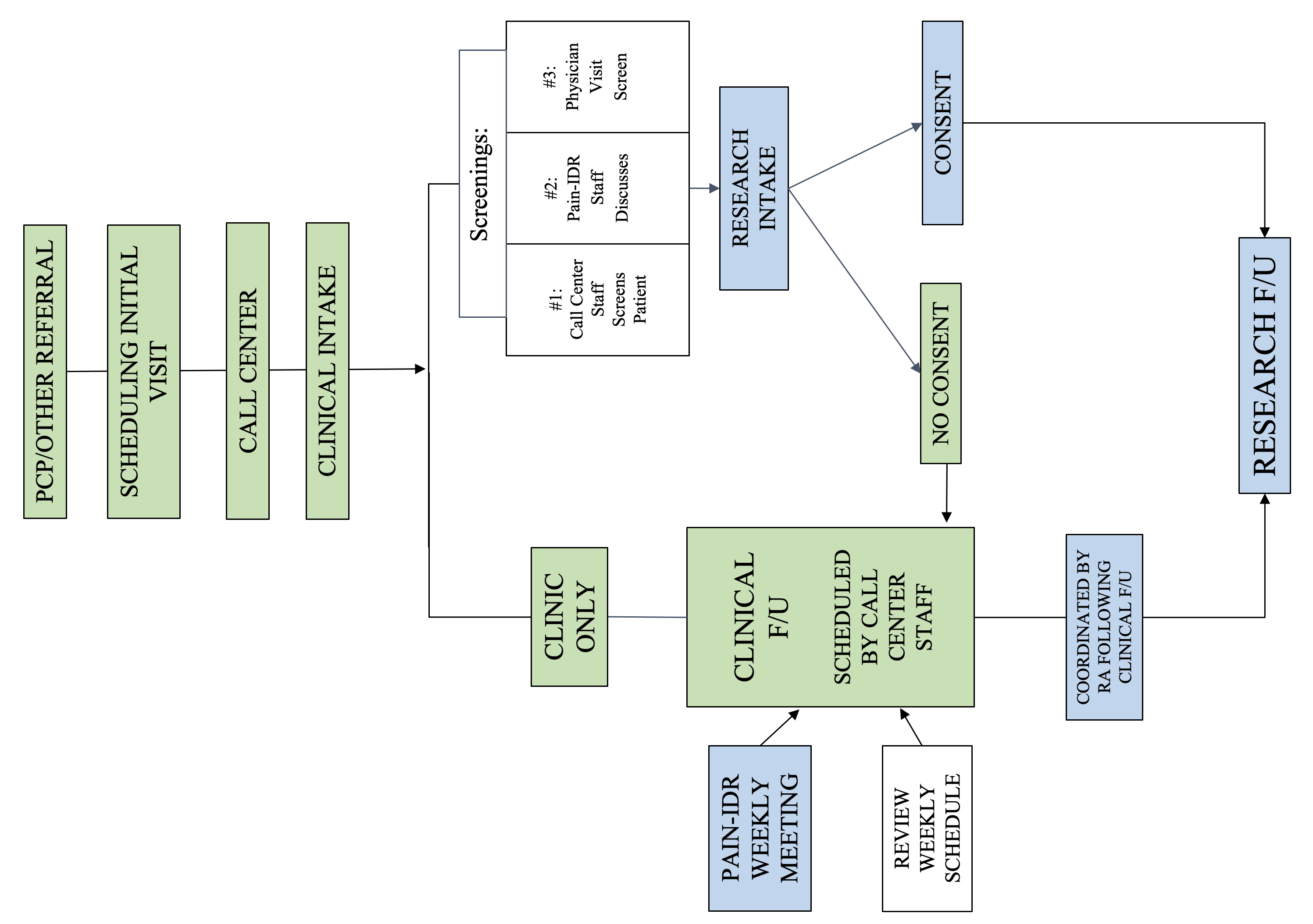
**

**Supplemental Table 1.** User Engagement Scale Short-Form (UES-SF) feedback survey administered to participants at the end of the study.

| Survey | Question | Response Scale |
| --- | --- | --- |
| Freeform | How strongly do you agree or disagree with this statement?  "I enjoyed using the Beiwe app and being a part of the study." | Strongly disagree  Disagree  Neither agree nor disagree  Agree  Strongly agree |
|  | Please select any specific aspects of the Beiwe app and/or research study that you enjoyed.  If there are no aspects that you enjoyed, please select "Not applicable". | Beiwe app design  Filling out surveys  Recording audio logs  Compensation  App support  Not applicable  Other |
|  | Please feel free to elaborate on any choices selected. | Free response |
|  | Please select any specific areas of the Beiwe app and/or research study that you think could be improved.  If there are no areas you think could be improved, please select "Not applicable". | Beiwe app design  Compensation  Troubleshooting: Not being able to access surveys  Troubleshooting: Phone screen showing up as black  Troubleshooting: Other app issues  App support  Not applicable  Other |
|  | Please feel free to elaborate on any choices selected. | Free response |
|  | Is there anything else you would like to share with us about your experience in this research study? | Free response |
| User Engagement Scale | I lost myself in this experience. | Strongly disagree  Disagree  Neither agree nor disagree  Agree  Strongly agree |
|  | The time I spent using the Beiwe app just slipped away. | Strongly disagree  Disagree  Neither agree nor disagree  Agree  Strongly agree |
|  | I was absorbed in the Beiwe app. | Strongly disagree  Disagree  Neither agree nor disagree  Agree  Strongly agree |
|  | I felt frustrated while using the Beiwe app. | Strongly disagree  Disagree  Neither agree nor disagree  Agree  Strongly agree |
|  | I found the Beiwe app confusing to use. | Strongly disagree  Disagree  Neither agree nor disagree  Agree  Strongly agree |
|  | Using the Beiwe app was taxing. | Strongly disagree  Disagree  Neither agree nor disagree  Agree  Strongly agree |
|  | The Beiwe app was attractive. | Strongly disagree  Disagree  Neither agree nor disagree  Agree  Strongly agree |
|  | The Beiwe app was aesthetically appealing. | Strongly disagree  Disagree  Neither agree nor disagree  Agree  Strongly agree |
|  | The Beiwe app appealed to my senses. | Strongly disagree  Disagree  Neither agree nor disagree  Agree  Strongly agree |
|  | Using the Beiwe app was worthwhile. | Strongly disagree  Disagree  Neither agree nor disagree  Agree  Strongly agree |
|  | My experience was rewarding. | Strongly disagree  Disagree  Neither agree nor disagree  Agree  Strongly agree |
|  | I felt interested in this experience. | Strongly disagree  Disagree  Neither agree nor disagree  Agree  Strongly agree |

**Supplementary Table 2.** Smartphone features collected as part of the HERMES phenotype, seeking to quantify functional status.

| **Domain** | **Method/**  **Tool** | **Summary Measure** | **Sample Rate** | **Unit** |
| --- | --- | --- | --- | --- |
| Patient Reported Outcome Measure | PROMIS-29 Micro-survey | Physical Function | Daily | - |
|  |  | Pain Interference/  Intensity | Daily | - |
|  |  | Sociability | Daily | - |
|  |  | Emotional Distress | Daily | - |
| GPS Location | * | Time at home | 0.5-1.0 Hz** | Minutes |
|  |  | Distance Travelled |  | Meters |
| Accelerometer/  Gyroscope | * | Mean Daily Steps | Acc: 0.5-1Hz Gyro: 10-1000Hz** | Locations |
|  |  | Rest-to-Moving Ratio |  | Ratio |
|  |  | Movement Energy |  | Minutes |
|  |  | Sleep |  | Minutes |
| Audio Data | Hume.AI | Prosody and Semantic Loadings | Compressed at 64Kbps | Variable Loadings |
| Call/Text Logs | * | In/Outgoing Calls/Texts | Daily | Counts |
|  | * | In/outgoing Call/Text length | Daily | Characters |
|  | * | Out-degree | Daily | Counts |

* See <https://github.com/onnela-lab/beiwe-backend/wiki/%5BResearchers%5D-Summary-Statistics>

**Supplementary Table 3.** Patient Compensation Scheme. Summary of monetary compensation based on active and passive data type. Compensation for active data collection (surveys and audio diaries) increases over time while passive data compensation does not.

| Month | Data Type | Compensation | Total Compensation |
| --- | --- | --- | --- |
| Month 1 | Active | $40 | $45 |
|  | Passive | $5 |  |
| Month 2 | Active | $45 | $50 |
|  | Passive | $5 |  |
| Month 3 | Active | $50 | $55 |
|  | Passive | $5 |  |
| Month 4 | Active | $55 | $60 |
|  | Passive | $5 |  |
| Month 5 | Active | $60 | $65 |
|  | Passive | $5 |  |
| Month 6 | Active | $65 | $70 |
|  | Passive | $5 |  |
| Total Compensation | | $345 | $345 |

**Supplementary Table 4.** Table describing sample size, study duration, adherence for surveys, adherence for passive data, and patient population for prior research conducted using the Beiwe digital phenotyping platform.

|  | **Sample Size** | **Study Duration** | **Adherence (Surveys)** | **Adherence (Accelerometer or GPS)** | **Patient population** |
| --- | --- | --- | --- | --- | --- |
| Torous et al 2015 | 13 | 29-30 days | 77.78% | Data type not collected | Major depressive disorder |
| Wright et al 2018 | 8 | 30 days | 70% | 90.00% (Accelerometer) | Gynecologic cancer, palliative chemotherapy |
| Barnett et al 2018 | 15 | Up to 3 months | No data available | No data available | Relapse |
| Straczkiewicz et al 2022 | 14 | 154 days (mean) | Data type not collected | 58.00% (Accelerometer), 46.00% (GPS) | Major depressive disorder, schizophrenia, or bipolar disorder |
| van den Berg et al 2022 | 95 | 131 days (mean) | 76.00% (Surveys at 4,12, and 24 weeks); 34.00% (Daily surveys) |  | Patients recovering from cancer surgery |

**Supplementary Table 5.**Table summarizes troubleshooting category, number of instances and percentage breakdowns, elucidating prevalence of specific issues.

| **TROUBLESHOOTING CATEGORY** | **NUMBER OF INSTANCES** | **PERCENTAGE** |
| --- | --- | --- |
| SURVEY NOT SHOWING | 6 | 12.2% |
| DATA UPLOADING | 16 | 32.7% |
| MIC ISSUES | 2 | 4.1% |
| MISCELLANEOUS | 15 | 30.6% |
| CONFLATED (MIC/DATA) | 2 | 4.1% |
| CONFLATED (MISC/DATA) | 1 | 2.0% |
| CONFLATED (SURVEY/DATA) | 1 | 2.0% |
| OTHER | 6 | 12.2% |
| **TOTAL TROUBLE SHOOTING** | 49 | 100.0% |
